# Cavernomatose cérébrale sporadique révélée par une crise convulsive: à propos d’un cas

**DOI:** 10.11604/pamj.2018.31.162.17052

**Published:** 2018-11-02

**Authors:** Doumbia Amadou, Koné Youssouf, Maïga Oumou, Koné Abdoulaye, Diarra Bréhima, Dembélé Adama, Diallo Mamahadou

**Affiliations:** 1 Service d’Imagerie Médicale, Centre de Santé de Référence de la Commune VI, Bamako, Mali; 2 Service Radiologie, Centre Hospitalier Régional Universitaire de Brest, France; 3 Service de Radiologie, Centre Hospitalier Jacques Boutard, France; 4 Service Radiologie, Centre Hospitalier Universitaire Gabriel Touré, Bamako, Mali; 5 Service d’Imagerie Médicale, Centre Hospitalier Universitaire de Yopougon, Côte d’Ivoire; 6 Centre de Radiologie Diagnostique et Interventionnelle, Bamako, Mali; 7 Service Radiologie, Centre Hospitalier Universitaire du Point G, Bamako, Mali

**Keywords:** Cavernomatose, convulsion, scanner, IRM, Cavernous malformation, convulsions, scanner, MRI

## Abstract

La cavernomatose cérébrale est une pathologie rare pouvant être sporadique ou familiale autosomique dominante. Elle est caractérisée par la présence de cavernomes multiples du système nerveux central. Souvent asymptomatique, la pathologie peut se révéler par des symptômes variés comme l’hémorragie cérébro-méningée, les céphalées ou l’épilepsie. Nous rapportons un cas de cavernomatose cérébrale sporadique chez un patient de 55 ans sans antécédent pathologique particulier révélé par une crise d’épilepsie. A travers cette observation et une revue de la littérature, nous faisons le point sur les aspects cliniques et radiologiques (scanner et IRM) de cette pathologie.

## Abstract (en)

Cerebral cavernous malformation is rare. It can occur sporadically or as a familial autosomal dominant disorder (FCCM). It is characterized by the presence of multiple cavernous malformations of the central nervous system. It is often asymptomatic and can be revealed by several symptoms including cerebro-meningeal hemorrhage, headaches or epilepsy. We report the case of a 55-year old patient, with no particular past medical history, with sporadic cerebral cavernous malformation revealed by seizure. Through this case study and literature review, we aim to highlight the clinical and radiological features (scanner and MRI) of this disease.

## Introduction

Les cavernomes représentent 1% de toutes les lésions vasculaires intracrâniennes et 15% de toutes les malformations vasculaires cérébrales [1]. Leur pic de fréquence se situe aux alentours de 20 à 40 ans mais ils peuvent se voir à tous les âges de la vie. Ils peuvent être uniques ou multiples, familiaux ou sporadiques [2]. La cavernomatose représente plus de 33% des cas. Le caractère familial est rare mais a été décrit tout comme la forme sporadique [2]. La transmission se fait selon un mode autosomique dominant à pénétrance variable [2]. Trois gènes ont été identifiés dans la littérature, ils codent pour des protéines impliquées dans l’angiogénèse à partir de l’endothélium vasculaire [3, 4]. Il s’agit des gènes KRIT1 (ou CCM1) sur le chromosome 7q; MGC4607 (ou CCM2) sur le chromosome 7p et PDCD10 (ou CCM3) sur le chromosome 3q [2-4]. Souvent asymptomatique, la pathologie peut se révéler par une hémorragie cérébro-méningée, des céphalées ou une épilepsie. Nous rapportons un cas de cavernomatose cérébrale sporadique révélée par une crise convulsive chez un patient de 55 ans.

## Patient et observation

**Figure 1:**
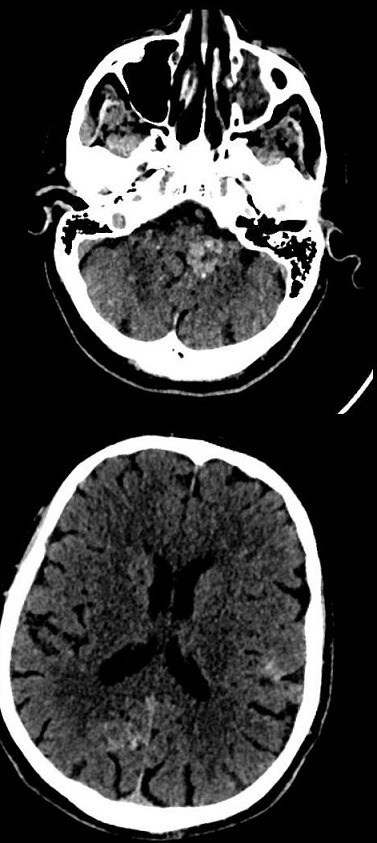
scanner encéphalique sans injection, coupe axiale en fenêtre parenchymateuse à l’étage infra tentoriel et supra tentoriel décelant plusieurs hyperdensités spontanées intra axiales

**Figure 2:**
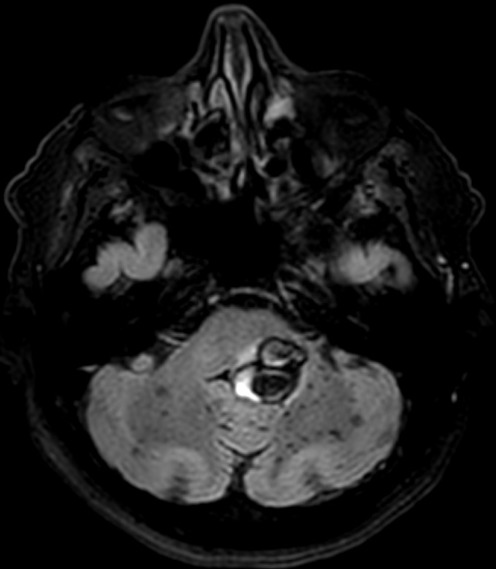
IRM cérébrale, coupe axiale à l’étage infra tentoriel, séquence T2 FLAIR montrant des cavernomes multiples en hyposignal hétérogène « en poivre et sel »

**Figure 3:**
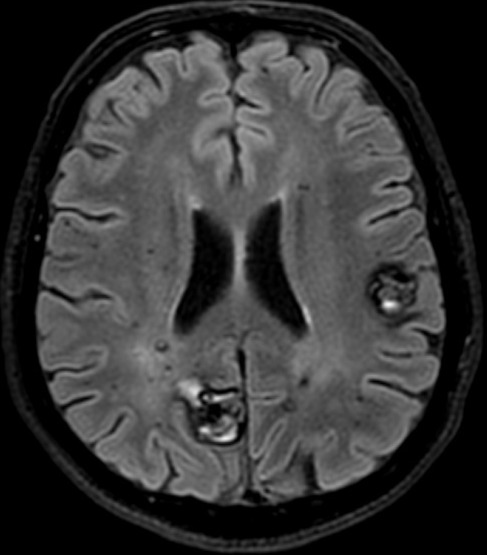
IRM cérébrale, coupe axiale à l’étage supra tentoriel, séquence T2 FLAIR montrant des cavernomes multiples en hyposignal hétérogène « en poivre et sel »

**Figure 4:**
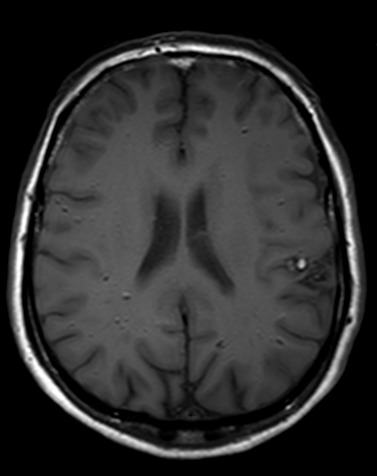
IRM cérébrale, coupe axiale à l’étage infra tentoriel avec de multiples cavernomes en discret hyper signal séquence T1

**Figure 5:**
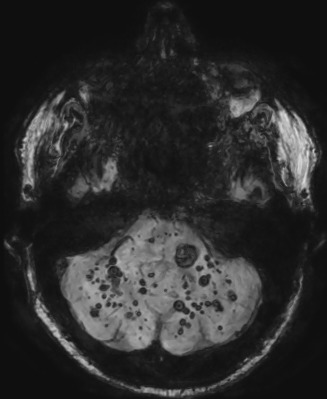
IRM cérébrale, coupe axiale en séquence T2 écho de gradient, multiples cavernomes en hyposignal franc

**Figure 6:**
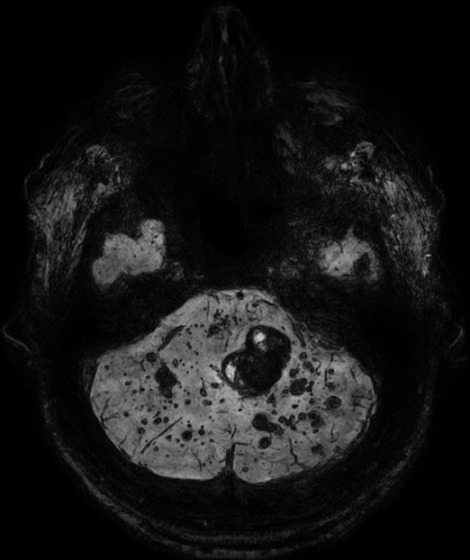
IRM cérébrale, coupe axiale en séquence de susceptibilité magnétique SWI montrant la cavernomatose

**Figure 7:**
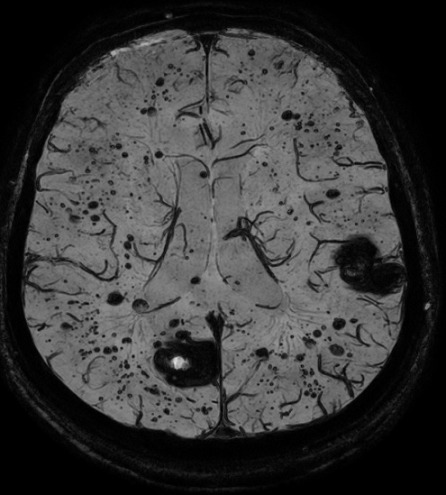
IRM cérébrale, coupe axiale en séquence de susceptibilité magnétique SWI mettant en évidence de multiples cavernomes en hyposignal franc

Il s’agit d’un patient de 55 ans sans antécédent particulier, qui présente au cours d'une réunion de travail un malaise sans perte de connaissance, suivi de crises convulsives tonico-cloniques. Il décrit également un déficit sensitif de l'hémicorps gauche totalement régressif. A l’examen d’entrée, il est apyrétique avec un score de Glasgow à 15, une pression artérielle à 130/80mmHg, une fréquence cardiaque à 70 battements par minute. Absence de déficit sentivo-moteur à l’examen neurologique et les réflexes ostéo-tendineux sont normaux. Pas de foyer épileptogène à l’électro-encéphalogramme et le bilan biologique était normal. Pas de facteurs de risque cardiovasculaire, le score NIHSS (National Institues of Health Stroke Scale) = 0. Le scanner cérébral sans injection objective plusieurs hyperdensités spontanées intra axiales, infra et supra tentorielles (Figure 1). L’IRM cérébrale confirme la présence de ces lésions intra-axiales qui sont en hyposignal hétérogène « en poivre et sel » T2 Flair (Figure 2 et Figure 3), en discret hyper signal en séquence pondérée T1 (Figure 4), en hyposignal franc couvrant la quasi-totalité des lésions en T2 écho de gradient (Figure 5) et en séquence SWI (Figure 6 et Figure 7). Elles sont entourées d’un liseré en hyposignal sur toutes les séquences. L’aspect IRM est caractéristique d’une cavernomatose cérébrale.

## Discussion

Les cavernomes cérébraux sont des malformations vasculaires veineuses qui représentent 5 à 10% de toutes les malformations vasculaires du système nerveux central [5]. Ils peuvent avoir un caractère sporadique ou familial. Dans les cas familiaux on note une fréquence plus élevée de cavernomes multiples par rapport aux cas sporadiques [2]. Chez notre patient l’enquête familiale n’a pas retrouvé de mutation génétique. Nous n’avons pas aussi retrouvé d’antécédent d’épilepsie dans sa famille notamment chez ses parents et ses enfants d’où l’hypothèse de cavernomatose cérébrale sporadique. Du point de vue clinique, les cavernomes peuvent être asymptomatiques ou se manifester par des crises convulsives dans 40 à 50% des cas. Les signes neurologiques focaux sont retrouvés dans 20% des cas. Les autres manifestations cliniques des cavernomes sont les hémorragies ou les céphalées [2]. L’hémorragie des cavernomes est le plus souvent silencieuse, mais elle peut être rarement massive et grave dans 0,25 à 6% des cas [5]. L’épilepsie est un autre symptôme de la maladie. Le risque de survenue de crises d’épilepsie est de 1,51% à 2,4 % par an et par malade [6]. Il semble qu’un âge d’apparition jeune, inférieur à 40 ans, soit un facteur de risque d’épilepsie pharmaco-résistante [6]. Chez notre patient âgé de 55 ans, la maladie a été révélée par une crise d’épilepsie. Dans la littérature, Reix G *et al.* [7] ont également rapporté une épilepsie révélant un cavernome cérébral familial chez une fille de 10 ans.

L’imagerie, notamment l’IRM, est incontournable dans le diagnostic de la cavernomatose. Au scanner, les cavernomes se présentent dans la plupart des cas sous forme d'hyperdensités inhomogènes dans 58,3% et se rehaussent après injection de produit de contraste dans 50%. Un discret effet de masse et un œdème péri-lésionnel n’étaient retrouvés que dans 4,16% par Broder M *et al.* [8]. Ces anomalies scanographiques des cavernomes ont été retrouvées chez notre patient. Dans notre observation, le scanner cérébral sans injection objectivait de multiples lésions arrondies intra parenchymateuses spontanément hyperdenses, infra et supratentorielles, hétérogènes sans œdème péri-lésionnel significatif. On observait un discret rehaussement des lésions cérébrales après injection de produit de contraste iodée. Selon Broder M *et al.* [8] les calcifications sont présentes dans 16,6% et témoignent des saignements intra lésionnels chroniques. L’anomalie de drainage veineux manquait dans notre observation au scanner contrairement à Broder M *et al.* [8] qui la retrouvait dans 25% des cas. Par ailleurs nous n’avons pas décelé de calcifications au sein des lésions cérébrales au scanner. Par contre chez notre patient on mettait en évidence de discrets remaniements hémorragiques aigus au sein de certains cavernomes pouvant expliquer la crise convulsive révélatrice.

L’IRM est l’examen de choix pour le diagnostic de la cavernomatose cérébrale [2, 5, 7, 9, 10]. Dans sa forme typique, un cavernome comporte une zone centrale hétérogène associant un hypersignal intense en T1 et T2 lié à la présence de méthémoglobine et un hyposignal dû au mélange calcium-hémosidérine avec une zone périphérique en hyposignal T2 (hémosidérine) [9, 10]. Il existe des formes atypiques lors d’hémorragie récente importante ou en cas de malformation vasculaire associée notamment un angiome veineux [10]. Chez notre patient, l’IRM confirmait le diagnostic de cavernomatose en mettant en évidence multiples lésions de taille et de topographie variables en hyposignal T2 hétérogène « aspect en poivre et sel », présentant un discret hypersignal sur la séquence pondérée en T1. Les séquences T2 écho de gradient et SWI (Susceptibility Weighted Imaging) mettaient en évidence de multiples cavernomes sous formes d’un hyposignal franc. On retrouvait aussi un liséré en hyposignal de la quasi-totalité des lésions sur toutes les séquences. L’aspect IRM des cavernomes de notre observation est en faveur d’une cavernomatose de type I de la classification de Zabramski *et al.* [11]. L’évolution clinique du patient était satisfaisante. L’IRM cérébrale de contrôle mettait en évidence une stabilité de la cavernomatose comparativement à l’examen initial.

## Conclusion

L’IRM est le meilleur examen pour le diagnostic de la cavernomatose cérébrale. Le risque hémorragique est la principale complication de cette pathologie. L’épilepsie est un mode de révélation possible de la maladie. L’enquête familiale est une étape importante afin de différencier une forme sporadique d’une cavernomatose familiale.

## Conflits d’intérêts

Les auteurs ne déclarent aucun conflit d’intérêts.

## References

[1] Gueddari FZ, Dafiri R, Imani F (1998). Apport de l’IRM au diagnostic des cavernomes intracrâniens. Médecine du Maghreb.

[2] Chabbchoub Ben Abdallah R, Kammoun F, Ayedi M, Trabelsi L, Ben Salah M, Ben Hlima N, Mahfoudh A (2010). Cavernomatose cérébrale chez une fille de 1 an. Arch Pediatr.

[3] Sempere-Pérez A, Campistol J, Garcia-Cazoria A (2007). Multiple familial cerebralcavernomatosis. Rev Neurol.

[4] Revencu N, Vikkula M (2006). Cerebral cavernous malformation: new molecular and clinical insights. J Med Genet.

[5] Avci E, Ozturk A, Baba F (2007). Huge cavernoma with massive intracerebral hemorrhage in a child. Turk Neurosurg.

[6] Pierre Labauge, Alice Lebayon (2004). Cavernomes cérébraux, histoire naturelle, facteurs aggravants Neurologies. Février.

[7] Reix G, Stoven C, darcel F, Gauthier-Lasalarié P, Plésiat-Trommsdorff V, Bintner M, Flodrops H (2009). Cavernome cérébral familial: révélation par une épilepsie chez une fille de 10 ans. Archives de Pédiatrie.

[8] Broder M, Maeder PH, de Tribolet N (2000). Angiomes caverneux et anomalies de drainage veineux cérébrales associées. Revue Médicale Suisse.

[9] Pinsart N, Arthus M, Pinsard N, Ponsot G (1998). Pathologie vasculaire. Neurologie pédiatrique.

[10] Gueddari FZ, Dafiri R, Imani F (1998). Apport de l’IRM au diagnostic des cavernomes intracrâniens. Médecine du Maghreb.

[11] Zabramski JM, Washer TM, Spetzler RF (1994). The natural history of familial cavernous malformations: results of an ongoingstudy. J Neurosurg.

